# Cadaveric Identification through Macroscopic Analysis of Dental Implants Subjected to High Temperatures—An Experimental Model

**DOI:** 10.3390/jfb14020107

**Published:** 2023-02-14

**Authors:** Ana Isabel Serrano-Esteban, Estefanía Requena-Gómez, Jesus Mena-Alvarez, Cinthia Rodríguez, María Bufalá-Pérez, Juan Manuel Aragoneses

**Affiliations:** 1 Department of Criminal Law and Criminology, International University of La Rioja (UNIR), 26006 Logroño, Spain; 2 Faculty of Dentistry, Catholic University of Valencia Saint Vicent Martyr, 46001 Valencia, Spain; 3 Faculty of Dentistry, Alfonso X El Sabio University, 28691 Madrid, Spain; 4 Department of Dentistry, Universidad Federico Henriquez y Carvajal, Santo Domingo 10106, Dominican Republic

**Keywords:** apical width, catastrophe, dental implants, identification, interspiral distance, temperature

## Abstract

The aim of this study was to determine the possibility of identifying a dental implant through the measurement of the apical width and the interspiral distance in a periapical radiograph after being subjected to high temperatures for certain lengths of time. In total, 11 fresh human anatomical models were selected, in which 137 implants were placed. Previous periapical radiographs were performed using parallelizers in each implant. Subsequently, the anatomical models were introduced into a crematory oven at different temperatures and for various durations: 500 °C/15 min, 500 °C/30 min, 700 °C/15 min, 800 °C/15 min, 800 °C/45 min, 500 °C/15 min, 700 °C/15 min, and finally, 1000 °C/120 min. After this, X-rays were taken via a parallel technique, and the apical width and interspiral distance were measured. The implants were disinserted, and the coronal width was used to calculate magnification or possible distortion. All data were analyzed by the Mann–Whitney U test. There were no statistically significant differences for the apical width parameter, except when the temperature was raised to 700 °C/15 min and to 800 °C/45 min. For the interspiral distance parameter, there were no statistically significant differences, except when the implants were subjected to 800 °C/15 min and 1000 °C/120 min. It was determined that there were changes in some groups based on the increase in temperature and exposure time. Neither of the two parameters were completely useful for the identification because some of the groups studied in both variables presented differences, which makes them difficult to identify correctly.

## 1. Introduction

The main objective of forensic pathology is to investigate skeletal bone remains in order to identify the subject [1]. The methods used for identification are different depending on whether it is carried out on living subjects, on recent corpses, or on skeletons and/or cadaveric remains [2].

In burned cadavers, identification can be a difficult task [3,4,5], since the bodies are too destroyed, and it is necessary to resort to necro-identification [5,6]. Dental tissue is the most durable [7] and helps not only in extreme cases of great deterioration of the structures but also in milder cases; further, this identification can be carried out simply, cheaply, and quickly, compared to other very reliable techniques such as the study of DNA [8]. In major catastrophes, members of the same family are often involved, which also makes it difficult to take DNA samples for identification.

The main problem for forensic identification lies in the adequate presence of ante-mortem clinical records in dental history, as described by Clark [9]. Previous medical histories must be properly completed, including dental history and previous X-rays. It is also important to facilitate identification work by having prostheses incorporate the name of the patient. The discrepancies are often due to errors in the registration of medical records, such as type of prosthesis, number of teeth, materials used [10].

Currently, there are several ways to identify subjects who have been subjected to the action of fire and heat, which causes major structural changes in human tissues, making the use of papilloscopy and other common methods of identification impossible [11]. However, teeth constitute an ideal target for investigation, identification, and necro-identification due to their special characteristics [12]. There is a drawback in the dental identification of patients subjected to heat, which is that the teeth can survive the action of temperatures of 1100 °C, but they can also be destroyed by high heat stroke [13].

It has been established that to incinerate an adult subject, temperatures ranging from 800 to 1200 °C are needed for 45 to 120 min depending on the size and water content [14], leaving from 1 to 3 kg of ashes. However, other authors have established that intervals of 90–150 min are necessary with temperatures around 1000 °C [15], while when it was carried out with gas engines, intervals of 60–90 min were necessary at 800 °C [16]. These intervals are supported by ABFO guidelines [17].

Dental implants lack the singularity that manual restorations have, since they are mass-produced [18], but the main advantage they present is their great resistance to mechanical and thermal agents [19,20]. In cases in which the teeth are subjected to extreme temperatures, it has been seen that the crowns of the teeth separate from the roots, thus allowing the evaporation of the pulp tissue [21] and preventing the use of the DNA present in this tissue to carry out molecular biology techniques [22]; however, this emphasizes the value of the genetic analysis of mineralized tooth tissues as an alternative to pulp, especially in extreme forensic conditions [23]. Various studies have shown that, in cases of severe incineration, the implant body and intermediate prosthetic abutment may be the only dental remains, as conventional dental materials, including amalgam, composite, and gold, can melt or distort [21,24,25]. Implants have a high melting point; for titanium, it is above 1650 °C [26], while for zirconium, it exceeds 1850 °C [27]. This physical property of the implants, together with the different designs, diameters, lengths, and surface treatments, could help identify victims, especially in cases where there is no other scientific evidence, such as DNA or fingerprints, and there is a loss of fragile dental remains [28].

Fire and high temperatures produce the destruction of a large part of the human body. Bones, being mainly composed of inorganic matter, are more resistant to alteration. The fact that the implants are inserted inside the bone and become perfectly attached to it through the phenomenon known as osseointegration means that they are especially protected against external agents. Implants add high resistance to physical agents; they thus become a rich source of information in cases of necro-identification, especially when other methods cannot be used, or as a complement to other techniques that are more expensive or require a long time to complete [29].

The aim of this study was to determine how implants at high temperatures behave with a view to their possible application in identification in ante-mortem/post-mortem comparison, as well as delving into the usefulness of implants in the field of forensic dentistry and necro-identification. We further sought to determine if it is possible to identify a dental implant through the measurement of the apical width and the distance between spirals in a periapical radiograph.

## 2. Materials and Methods

A single-blind, randomized, experimental in vitro post-mortem study was conducted in accordance with the principles defined in the statement of the German Ethics Committee for the use of organic tissues in medical research (Zentrale Ethikkommission, 2003) and was approved by the University Ethics Committee (Process No. 10/1/2012). For each patient, the family gave their informed consent to transfer the body for this study. In total, 11 human anatomical models were used, and 137 dental implants with different macroscopic design, geometry, length, spire design, interspiral distance, coronal and apical diameters, and length were placed, with three different compositions: grade IV pure titanium, titanium–aluminum–vanadium (grade V titanium), and zirconium oxide ceramics. The randomization of the study sample was carried out using EPIDAT 4.2 (Dirección Xéral de Saude Pública, Galicia, Spain). Inclusion and exclusion criteria are reflected in Table 1.

**Figure 1:**
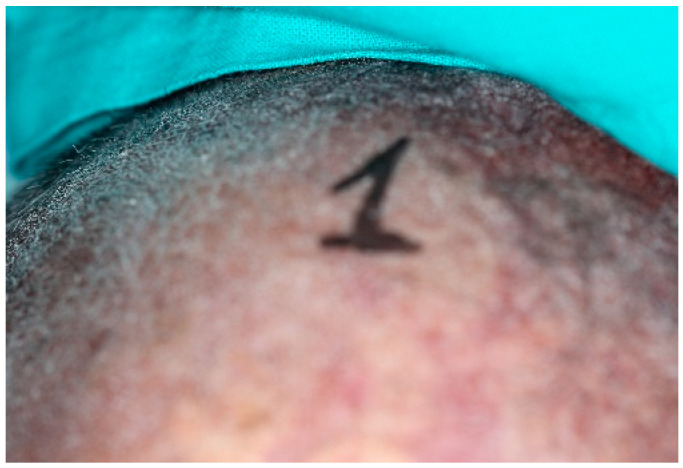
Anatomical model marking.

Prior to the placement of the dental implants, the anatomical models were divided numerically, being marked from 1 to 11 by means of a tattoo with Chinese ink at the level of the frontal area of the face (Figure 1). The assignment of the numbers was random, by awarding each anatomical model an envelope containing a number from 1 to 11.

The different measurements of the implants were taken using a correctly calibrated digital vernier caliper (Stainless Hardened^®^). These measurements were of the length, coronal diameter, diameter of the first thread, diameter of the last thread, distance between threads, thread design, diameter of the surface of the implant body, apical diameter, height of the polished neck, design and diameter of the platform, and prosthodontic connection. Although all these parameters or variables were recorded, only the interspiral distance and the apical width were studied.

Dental implants were inserted in various locations in the upper and lower jaws, using the standard drilling surgical protocol for edentulous sockets and for immediate post-extraction implants. The main requirement was that the models be partially edentulous, to be able to place dental implants in dental absences, as is done in traditional surgery. Post-extraction implants were also performed in those models in areas that were considered suitable for this technique. After placing the dental implants in the different anatomical models, dental X-rays of the implants were taken using the parallel or long-cone technique with the use of parallelizers (XCP-Rinn^®^, Dentsply Sirona, Ballaigues, Switzerland). Once the dental implants were placed, the anatomical models were taken to a crematorium where they were subjected to the cremation process at different times and temperatures, as follows. Group 1: implants exposed to 500 °C for 15 min; Group 2: implants subjected to 500 °C for 30 min; Group 3: implants exposed to 700 °C for 15 min; Group 4: implants exposed to 800 °C for 15 min; Group 5: implants subjected to 800 °C for 45 min; Group 6: implants subjected to 500 °C for 15 min and subsequently to 700 °C for another 15 min; Group 7: implants recovered after total cremation of the anatomical models at 1000 °C for 2 h (Table 2).

**Figure 2:**
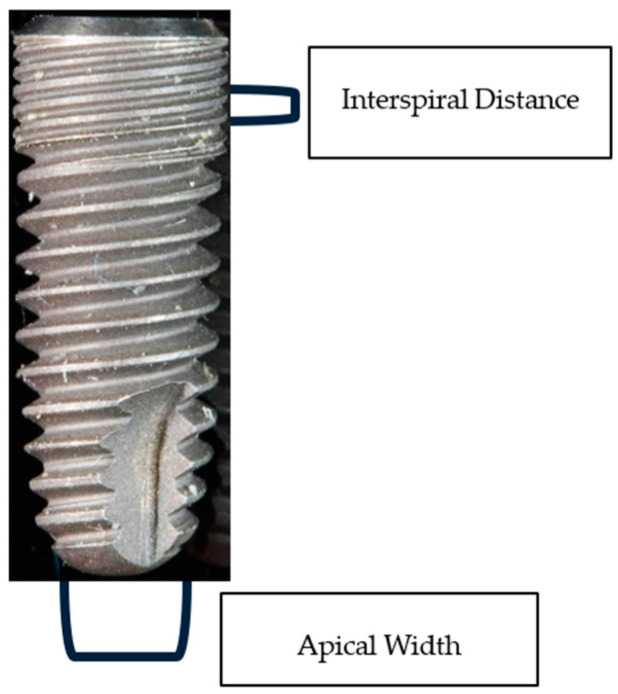
Implant representation of apical width and interspiral distance.

After being subjected to high temperatures, the anatomical models were X-rayed via the parallel technique. Through a computer program, different variables were measured with the chosen parameters being the apical width and interspiral distance. Implants were disinserted, and the real coronal width was measured using a digital caliper; this allowed the calculation of magnification or possible distortion, which was then extrapolated to the rest of the parameters (Figure 2). Not much is described by these parameters; what we wanted to see with this hypothesis was whether it was feasible to identify the patients through said variables.

An oven was selected that would allow for high temperatures to be reached in a controlled manner (Lazar, Atroesa^®^). Before introducing the anatomical models into the oven, the oven was preheated for two hours to obtain the initial agreed temperature of the project. The temperature of the chamber was evaluated by means of a thermocouple (temperature sensor) located in the center of the ceiling, and data could be obtained through an electronic temperature controller located outside.

The designation of the temperatures and times to which the anatomical models were going to be subjected was not random; different conditions were taken into account: titanium’s resistance to corrosion substantially decreases from 649 °C, [30] so a higher temperature (700 °C) was selected to determine whether oxidation could affect the macroscopic design of the implants, and a lower one (500 °C) was selected to determine if, indeed, no change occurred in the macroscopic structure of the implants due to phase change. Before the total decomposition of the bones and teeth, which takes place above 1000 °C, a lower temperature (800 °C) was selected. At this temperature, the reduction in the volume of the dental roots begins [31], and the dental crown is usually already devastated, so it was interesting to evaluate the effect of this temperature on the dental implants. Total incineration reducing to ashes is reached at temperatures of at least 1000 °C for more than two hours, so this was the maximum temperature and time recorded in this study.

The anatomical models were subjected to different temperatures in the oven, so X-rays of the dental implants were taken in association with the different times and temperatures to assess the final condition of the implants, as well as possible changes in the substructure. The parallel technique described above was used. Some models did not retain their integrity, so radiographs of the existing loose bone fragments were made without a parallelizer by placing the focus 10 mm from the plate in an attempt to identify the dental implants by their position, their morphology, and the remaining bone found. The radiological study was completed by means of a photographic protocol determined at the beginning of the study to compare these photographs with the previous ones and assess possible macroscopic changes.

When the anatomical models were subjected to total calcination, they were reduced to ashes, detaching the implants from the bone. The dental implants were found inside the crematorium oven and were recovered for study, classifying them into groups according to their length and diameter and measured using the digital vernier caliper. We evaluated whether it was possible to identify the implant despite not knowing its position or to which anatomical model it belonged. Dental implants were classified into different groups regarding the choice of implant, and X-rays of the removed implants were taken.

Once all the data had been collected and the radiographs had been digitally processed (TIFF), the pre- and post-incineration radiographic measurements were evaluated to assess whether there were dimensional changes using Adobe Photoshop software CS6^®^; these data were evaluated by statistical analysis. A supervisor, unrelated to the study, made the initial and subsequent measurements of all the records. The statistical study included comparisons of the apical width and interspiral distance using the IBM SPSS program (version 19) with a significance level of 5%. Since all the comparisons were made on pairs of data and it was detected that most of the groups did not follow the normal probability distribution, it was decided to apply the Mann–Whitney U test with the Bonferroni correction factor. 

## 3. Results

The records of the present study included the apical width and interspiral distance before and after being subjected to high temperatures, as determined from actual measurements of the implants and through radiographs. A posteriori, the magnification index was calculated and applied to the different records. Different study groups were distinguished based on the temperature and time exposed (Table 3).

**Figure 3:**
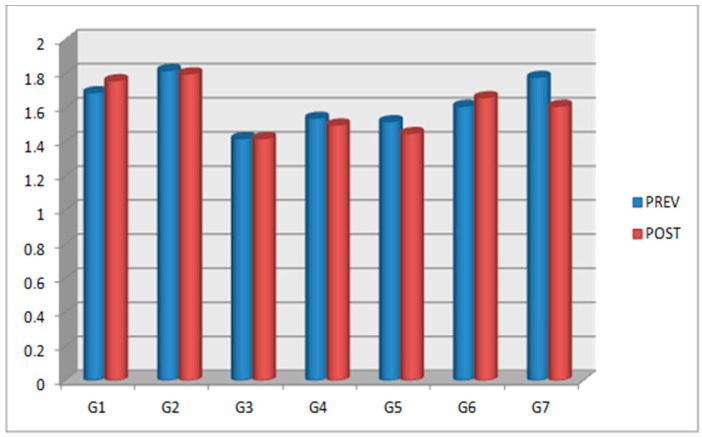
A summary of the groups’ average apical width values: previous (PREV) and posterior (POST).

**Figure 4:**
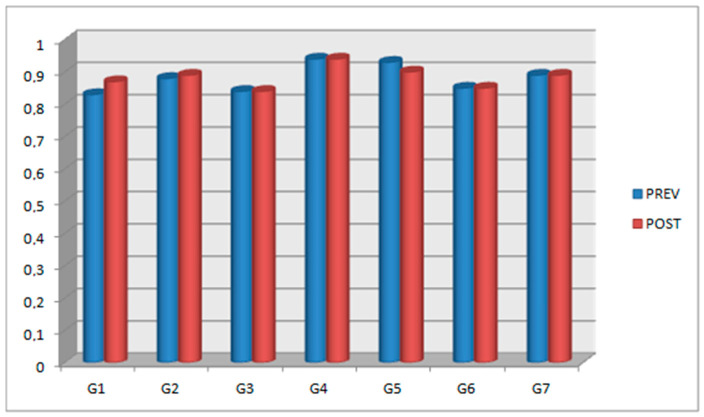
A summary of the groups’ average interspiral distance values: previous (PREV) and posterior (POST).

**Figure 5:**
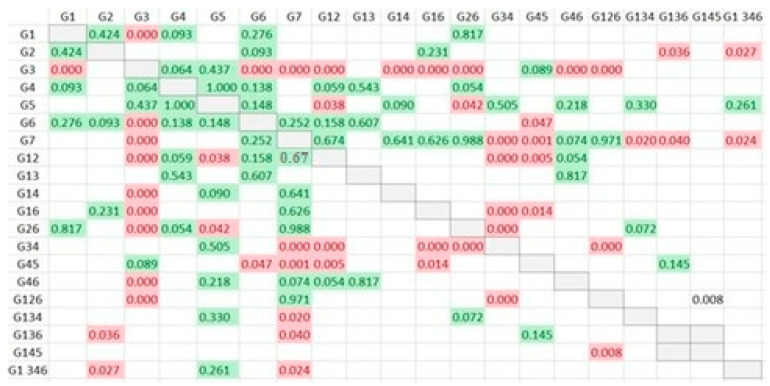
A summary of comparisons of the apical width, the value of the cells corresponds to p-Value (Red: statistically significant differences; green: no statistically significant differences).

**Figure 6:**
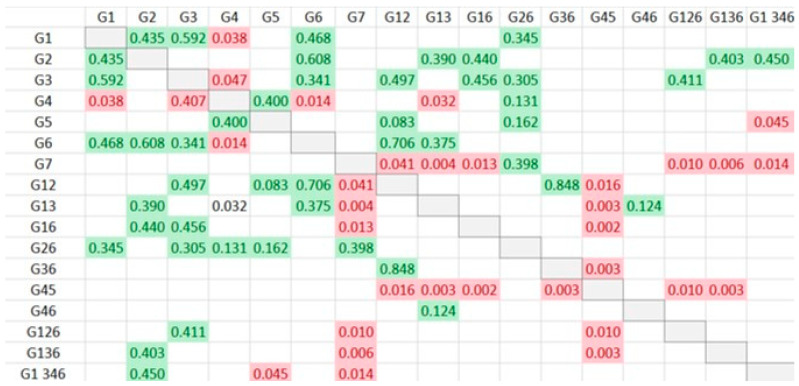
A summary of comparisons of the interspiral distance, the value of the cells corresponds to p-Value (Red: statistically significant differences; green: no statistically significant differences).

In Section 2, we described how a researcher outside the study recorded all the data before and after the anatomical models were subjected to high temperatures to avoid bias. As there were no statistically significant differences between the values for Group 1 (500 °C/15 min) before and after being subjected to high temperature, Group 1 was taken as the control group. A summary of the mean values of the groups’ apical width and interspiral distance (previous–posterior) is shown in Figure 3, Figure 4, Figure 5 and Figure 6.

Regarding the effect of the increase in exposure temperature on the apical width, there were statistically significant differences (*p* = 0.000) when 700 °C was applied for a maximum of 15 min. For the interspiral distance, there were statistically significant differences (*p* = 0.032) up to 800 °C for 15 min, after which dimensional changes were observed that could alter the measurement of the interspiral distance.

Regarding the effect of time for which dental implants were subjected to high temperatures on the apical width, there were statistically significant differences with an increase in time up to 45 min at a temperature of 800 °C (*p* = 0.041); however, no differences were observed at 500 °C or 700 °C, nor for 2 h at 1000 °C. Meanwhile, regarding the interspiral distance, there were no statistically significant differences with an increase in time up to 45 min at a temperature of 800 °C, nor were any found at 500 °C or for 2 h at 1000 °C (*p* > 0.05).

In order to avoid bias or errors in this study, the data after subjecting the dental implants to 500 °C for 15 min were taken as a reference and compared with the previous data for the same implants in the rest of the groups. For the apical width variable, Group 2 (implants exposed to 500 °C for 30 min), Group 4 (implants exposed to 800 °C for 15 min), Group 6 (implants exposed to 500 °C for 15 min and subsequently at 700 °C for another 15 min), and Group 7 (implants recovered after total cremation of the anatomical models at 1000 °C for 2 h) did not present statistically significant differences; therefore, they did not undergo modification and were still useful for identification. For the interspiral distance variable, Group 2 (implants exposed to 500 °C for 30 min) did not present statistically significant differences.

The combination of temperature and time revealed no statistically significant differences in the apical width, except when the temperature was raised to 700 °C for 15 min and to 800 °C for 45 min. For the rest of the temperatures and times, no differences were observed, neither when the temperature rose from 500 °C to 800 °C for 15 min, nor when it rose from 500 °C to 1000 °C for 15 min to 120 min, nor when it rose from 800 °C to 1000 °C for 45 to 120 min. No statistically significant differences were found in the interspiral distance, except when the implants were subjected to 800 °C for 15 min or when they were recovered from total calcination for two hours. There were no statistically significant changes when the temperature and exposure time were raised from 500 °C to 700 °C, with an interval of 15 to 30 min, but statistically significant differences were found at 2 h and 1000 °C. By analyzing all the recorded data and combining the two variables, it can be determined that there were changes depending on the increase in temperature and exposure time; however, there could be no correlation, and the changes could be due to radiographic distortion.

## 4. Discussion

This study was carried out to evaluate, at a macroscopic level and by means of dental radiography, whether there are pre- and post-mortem dimensional changes in dental implants subjected to high temperatures and whether it is possible to identify them. A first phase of the project was carried out, which included performing periapical radiographs via the parallel technique in the different stages of implant treatment. Once the implant was identified radiographically, it was extracted, and dental photographs were taken following the same previous photographic protocol. Through this process, we assessed whether it was feasible to identify the carriers of dental implants subjected to high temperatures with greater accuracy. Critical to this study was extensive research comparing post-mortem and pre-mortem data by forensic dentists. The clinical histories must be complete, but on many occasions, important and necessary data in the identification are lacking [11]. On the other hand, dental radiology is essential for identification; it is one of the most-used ante-mortem tests by dentists, and it is inexpensive, simple, and fast [28,32]. 

The macroscopic design of the implants at the radiographic level can favor the identification of the subjects, as well as the creation of a dental profile, as it allows for reducing the search range and simplifying it [33,34,35]. Human identification has been studied for decades and has been gradually advancing. Rezwana et al. used Adobe Photoshop 8.0 software in their study to compare photos of palatal roughness in patient identification [36], as in the present study, where measurements were also made through this software; however, the same system was not used. Here, we superimposed previous and post photographic images to identify the implants, but with the level of magnification calculated in this study to validate the research.

To incinerate an adult subject, temperatures ranging from 800 °C to 1200 °C for 45 to 120 min are required [14]; however, other authors have established that an hour and a half to two and a half hours is necessary with temperatures of around 1000 °C [15], while when carried out with gas engines, it takes from an hour to an hour and a half at 800 °C [16]. The use of dental implants for oral rehabilitation is a technique that has been established worldwide and that can serve as an identification method due to the superimposition of images and other procedures [28]. Implants lack the individuality of hand-made restorations, but their main advantage is their high resistance to mechanical and thermal agents [19,20]. This advantage was demonstrated in this work, since after the anatomical models were subjected to two hours of high temperatures, only the dental implants remained.

Berketa et al. conducted one study on dental implants in which they compared radiographs before and after the implants were subjected to high temperatures. The implants were subjected to high temperatures in an oven without any support mechanism, in contrast to this work, where the implants were housed inside the jaws of human anatomical models [18]. The present study does not coincide with previous results regarding the exposure time to which the implants were subjected; in Berketa’s study, they observed small alterations above 1125 °C that would not affect the identification of the implants. On the contrary, in the present study, in which seven groups were classified, radiographic alterations were observed in the implants regarding the apical width and interspiral distance in relation to the different temperatures and exposure times. Unlike Berketa’s study, in which the changes at a maximum temperature were mainly assessed, in this study, changes for intermediate times and temperatures were also assessed, with observations of statistically significant differences in some variables. We expect that these differences are probably due to the projection with which the radiography was made. Unlike the previous study, in which the projection was very exact and the implant was also isolated, in this study, the implants were inserted inside the jaws, which made it difficult to perform radiographs with the same projection, despite the use of parallelizers. Therefore, small variations or distortions can be observed in the radiographs, which could alter the measurements and, therefore, the results, potentially being the cause of the statistically significant differences in the two variables taken as a study guide. Statistically significant differences were observed in both variables, so based on these measurements, identification according to exposure time and temperature, as has been developed, may be difficult. This study is more in line with reality since it was carried out on human anatomical models and by matching pre-burned and post-burned radiographs, as would occur in conditions of major catastrophes, accidents, etc. 

Berketa et al. carried out a study in which post-extraction implants of pure titanium and TiAl4V alloy were placed in the sockets of the lower incisors of two heads of lamb and were placed in a crematory oven at 780 °C for two and a half hours. Their results were only close to the characteristics of Group 5 (800 °C/45 min) of the present study; therefore, the results are not comparable, although they determined that identification could be carried out in the implants when comparing pre-mortem and post-mortem [37]. For the apical width, our results were similar to those of Berketa, with no statistically significant differences observed in Group 4 at 800 °C for 15 min; this is useful for the future identification of implants via the comparison of pre-mortem data with post-mortem data. However, for this same group, whose characteristics were the most similar to those in the Berketa study, there were statistically significant differences for the interspiral distance, which prevents cadaveric identification and presents dissimilarities to Berketa.

The identification of dental implants would be easier if they had a serial or batch number on them, resistant to both chemical and physical agents. As of 2010, Straumann^®^ Corporation laser engraves the lot number on the inside of the connection of some of its implants. There is a large number of implants with the same batch number, varying between 24 and 2400. This number is very high, but compared to those that do not have a batch number, the possibilities of identification are increased in many cases [28]. In 2010, Berketa et al. exposed Straumann^®^ implants with engravings to 1125° for 5 min [28], showing that despite the high temperatures, the integrity of the internal engraving of the implant was maintained. However, the presence of a healing abutment is essential to allow pre-mortem and post-mortem comparisons. For the identification system to work, the dentist must have the batch number registered, and in this way, the subject could be identified [28].

The radiological method presents high validity, which has been evidenced by different experts in necro-identification, with positive results reaching 93% [38]. For this reason, we decided to use radiology to carry out this study and to try to find a method to identify implants that have been subjected to high temperatures. The results indicate that neither of the two variables would be 100% reliable for use in identification of the implants; as mentioned above, some of the experimental groups in this study presented statistically significant differences with regard to the apical width and the interspiral distance. This may be because despite using parallelizers, when the implants are subjected to high temperatures, changes are produced in the tissues due to calcination, resulting in changes in the radiological projection. The projection angle of the radiograph can influence the distortion present in the radiographs; the use of parallelizers avoids variations as much as possible, homogenizing pre-mortem and postmortem radiographs, as well as simplifying and improving the process. Therefore, the apical width and the interspiral distance are not useful to identify dental implants, so further studies with different variables or methods will be necessary to be able to identify dental implants without reservation. There are implants that have special characteristics, allowing their recognition quickly and easily, but there are other characteristics that make interpretation difficult or can even deceive [39]. In the present study, unique characteristics were not evaluated, only the dimensional changes in the interspiral distance and the apical width, with the conclusion that neither of these values is useful for identification due to the statistically significant differences observed in different groups. Other values should be studied in the future to expand upon this investigation and improve it. In addition, dentists must be familiar with the different implants, and there should be a database that allows identification [40].

The most important limitation of this study is that the implants were not osseointegrated, since this was a study that aimed to assess how the implants behaved at different temperatures and if there were differences between the titanium and zirconium implants. There are two main difficulties in the process of necro-identification by means of dental implants. One is the geometric projection of intraoral radiography, since it has been observed that changes in angulation cause distortion and magnification of the image, which can lead to false identification of the implant [41]. This inconvenience can be corrected with parallelizers, which were used in this study, taking into account the correct angle (distance and geometry) and the exposure time, although this technique does not ensure the correct size of the image. The other is the large number of implant systems with different designs that exist; in 2010, it was estimated that about 460 different types of implants exist [28]. Sahiwal et al. demonstrated that when vertical inclinations are greater than 10 degrees, greater distortion is produced, whereby the apical holes present in different implants will appear oblique and the shape of the threads also differs from the reality, making their identification more and more complicated [42]. In the present study, we observed that there were different groups that did not present distortion, but there was a lack of unanimity among all the groups; that is, there was a possibility of distortion and, therefore, difficulty in identification. This means that the study variables, the apical width, and interspiral distance are not valid parameters for identification. In this work, the distortion of the radiographs was corrected by calculating the ratio between both measurements (real post-burn and post-burn radiographs), which provided a numerical quantity to the magnitude of variation between the measurement obtained in the radiographic plate and the real measurement of the implant for later application to the rest of the variables. However, despite performing these calculations to control for distortion, statistically significant differences were observed, which makes identification difficult. In this study, we assessed the effect of high temperatures on the apical width and the interspiral distance, parameters measured on radiographs taken before and after exposure, from which it can be concluded that neither of the variables were totally useful for identification. Some of the groups studied presented statistically significant differences in both variables, which makes correct identification difficult.

## 5. Conclusions

Within the limitations of this study, we can conclude that the apical width of the dental implants recorded in the periapical radiographs was modified at temperatures of 700° for 15 min and 800° for 45 min, making it difficult to identify the implants. The interspiral distance was not affected by the increase in temperature when the implant was subjected to 700° C and did not undergo dimensional changes, so it can be determined that it did not hinder the interpretation or identification of these dental implants; however, from 800 °C, it lost its usefulness for identification. None of the variables were totally useful for identification, since some of the groups studied presented statistically significant differences in both variables, making it difficult to identify the implants correctly. However, these variations or distortions may have been due to the radiographic projection.

## Figures and Tables

**Table 1:** Inclusion and exclusion criteria.

Inclusion Criteria	Exclusion Criteria
Partially edentulous human anatomical models.	Fully dentate or edentulous human anatomical models.
Bone height of greater than or equal to 8 mm in any area, except in posterior maxillary areas.	Bone height of less than 8 mm in any location except in the posterior maxillary areas.
A minimum bone height of 6 mm in posterior maxillary areas.	Bone height of less than 6 mm in posterior maxillary areas.
Bone width of greater than or equal to 5.5 mm at any location.	Bone width of less than 5.5 mm at any location.
Existence of at least 2 mm of bone on each side of the implant, 1 mm vestibular, and another lingual or palatal.	Absence of at least 2 mm of bone on each side of the implant, 1 mm vestibular, and another lingual or palatal.
Primary stability of implants.	Absence of primary stability of the implants.
Implants placed juxtacrestally with respect to the vestibular cortical.	Implants placed supra or infracrestally with respect to the vestibular cortical.
Three-dimensionally correct implants surrounded by bone.	Three-dimensionally incorrect implants surrounded by bone.

**Table 2:** Study groups based on the temperature and time exposed.

Group	Temperature	Time	Number of Implants
1	500	15 min	40
2	500	30 min	14
3	700	15 min	16
4	800	15 min	9
5	800	45 min	11
6	500 + 700	15 min + 15 min	16
7	1000	120 min	31

**Table 3:** Temperatures and times in the different groups and number of implants per model.

Model	500 °C/30 min	800 °C/45 min	1000 °C/120 min	500 °C/15 min	700 °C/15 min	800 °C/15 min	Number of Implants
A	x		x				6
B		x	x				15
C		x	x				9
D	x			x			17
E	x		x	x			12
F			x	x	x		15
G			x	x	x	x	11
H					x		17
I			x		x		9
J						x	9
K			x			x	17

## Data Availability

The datasets used and/or analyzed during the current study are available from the corresponding author on reasonable request.
